# Co-intercalated layered double hydroxides as thermal and photo-oxidation stabilizers for polypropylene

**DOI:** 10.3762/bjnano.9.277

**Published:** 2018-12-05

**Authors:** Qian Zhang, Qiyu Gu, Fabrice Leroux, Pinggui Tang, Dianqing Li, Yongjun Feng

**Affiliations:** 1 State Key Laboratory of Chemical Resource Engineering, Beijing Engineering Center for Hierarchical Catalysts, Beijing University of Chemical Technology, No. 15 Beisanhuan East Road, Beijing 100029, China; 2 Université Clermont Auvergne, Institut de Chimie de Clermont-Ferrand ICCF, UMR-CNRS 6296, F 63171 Aubière, France

**Keywords:** co-intercalation, composites, layered double hydroxides, photo-oxidation stability, polypropylene, thermal stability

## Abstract

An elegant and efficient approach consisting in the co-intercalation of stabilizing molecular anions is described here. The thermal stabilizer calcium diethyl bis[[[3,5-bis(1,1-dimethylethyl)-4-hydroxyphenyl]methyl]phosphonate] (Irganox 1425, MP-Ca) and a photo-oxidation stabilizer (hindered amine light stabilizer, HALS) are co-intercalated into the interlayer regions of layered double hydroxides (LDH) in a one-step coprecipitation. These hybrid organic–inorganic materials are successively dispersed in polypropylene to form H*_n_*M*_n_*_′_-Ca_2_Al/PP composite films (with H = HALS and M = MP) through a solvent casting method. The corresponding crystalline structure, chemical composition, morphology as well as the resistance against thermal aging and photo-oxidation are carefully investigated by various techniques. The results show that the powdered H*_n_*M*_n_*_′_-Ca_2_Al-LDHs hybrid materials have a much higher thermal stability than MP-Ca and HALS before intercalation. In addition, the H*_n_*M*_n_*_′_-Ca_2_Al/PP composites exhibit a higher overall resistance against thermal degradation and photo-oxidation compared to LDHs intercalated with only HALS or MP. This underlines the benefit of the co-intercalation. The co-intercalated LDH materials pave a new way in designing and fabricating high-performance multifunctional additives for polymers.

## Introduction

Hindered phenols and hindered amines, containing the functional groups 2,6-di-*tert*-butylphenol and 2,2,6,6-tetramethylpiperidine, respectively, are widely used as functional additives in polymers to prolong the service life [1–3]. Generally, the anti-aging agents effectively inhibit the degradation in two ways: (1) through capturing generated free radicals and stopping auto-oxidation and (2) through decomposing and eliminating hydroperoxides [4–5]. However, anti-aging agents are often organic chemicals that easily migrate and volatilize from the polymer, reducing the anti-aging efficiency and increasing environmental pollution [6]. Therefore, it is of interest to explore novel multifunctional additives for polymers with high anti-aging performance together with high migration resistance.

**Figure 1:**
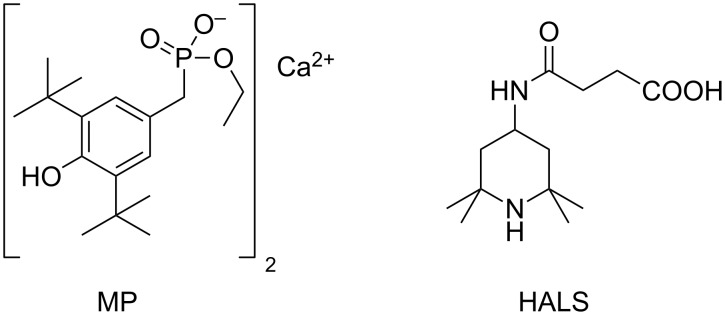
Molecular structures of Irganox 1425 (MP-Ca) and hindered amine light stabilizer (HALS).

Recently, inorganic–organic hybrid functional additives have attracted increasing attention for their wide applications in polymers [7]. Organic anti-aging species have been immobilized onto inorganic supports (e.g., carbon nanotubes, SiO_2_, graphene oxide) to produce inorganic–organic composites with higher migration resistance [8–10]. More recently, layered double hydroxides (LDHs), a layered host–guest material, have emerged as promising inorganic nanocontainers for functional organic active species to enhance the thermal and photo-oxidation stability of interleaved organic species as well as to endow the polymer/LDH composites with the desired properties [11–14]. In our previous work, a series of intercalated antioxidants and photo-oxidation stabilizers with a single active component have been prepared by coprecipitation. In these polymer/LDH compounds, the resistance against aging was significantly improved [15–17]. For example, the antioxidant Irganox 1425 (see Figure 1, abbreviated as MP-Ca) was intercalated into Ca_2_Al-LDH through coprecipitation of MP-Ca and Al(NO_3_)_3_ at pH 10, to yield MP-Ca_2_Al-LDH. Here, the MP-Ca was used the source of Ca for the host sheet and that of MP for the guest anions. Polypropylene (PP) protected with the prepared MP-Ca_2_Al-LDH exhibited enhanced thermal stability and anti-migration behavior in comparison with MP-Ca/PP composites. Lately, some studies have demonstrated much better performance of multi-component intercalation compounds compared to the corresponding single-component intercalation compounds as well as to the physical mixtures of the components [18–19]. The benefit of the co-intercalation is attributed to synergistic effects between the different active species associated to a higher dispersion in the composites [20–21].

In this work, we designed and fabricated a series of novel co-intercalated thermal and photo-oxidation stabilizers (H*_n_*M*_n_*_′_-Ca_2_Al) through straightforward co-precipitation of HALS and MP-Ca (Figure 1) [16–17], and examined the resistance of the H*_n_*M*_n_*_′_-Ca_2_Al/PP composites against thermal degradation and photo-oxidation as a function of the molar ratio between HALS and MP in the interlayer regions.

## Results and Discussion

### Analysis of H*_n_*M*_n_*_′_-Ca_2_Al-LDHs

**Figure 2:**
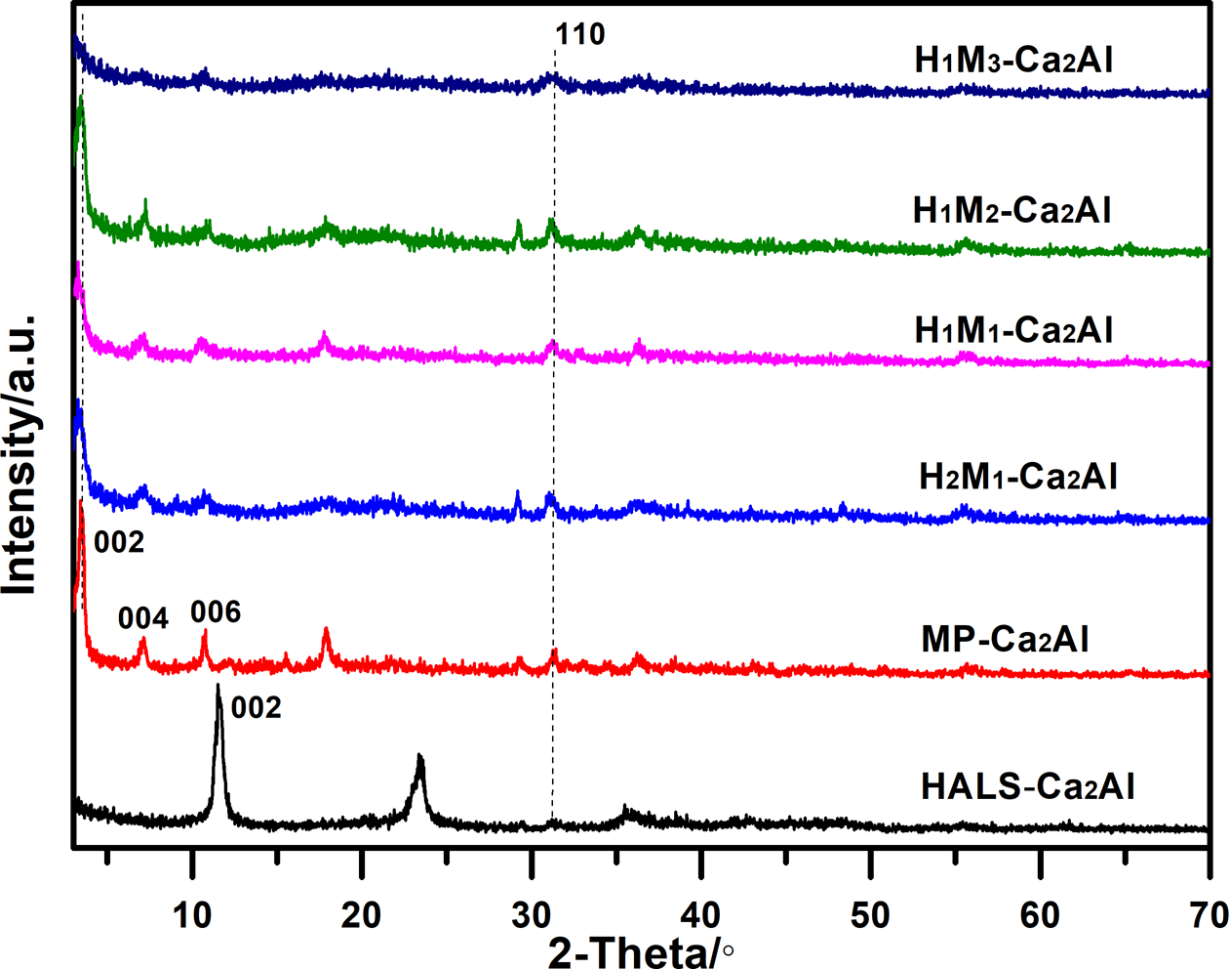
Powder X-ray diffraction patterns of different H*_n_*M*_n_*_′_-Ca_2_Al-LDH samples.

Figure 2 displays powder XRD patterns of H*_n_*M*_n_*_′_-Ca_2_Al-LDHs with sharp (002), (004) and (006) reflection peaks at low angles and the weaker (110) peak at a higher angle, corresponding to the layered structure and the intra-layer structure in the host sheet [22]. The (002) reflection peaks of HALS-Ca_2_Al and MP-Ca_2_Al are located at 11.5° (*d*_002_ = 0.77 nm) and 3.4° (*d*_002_ = 2.52 nm), respectively. Simultaneously, for LDHs co-intercalated with HALS and MP (H_2_M_1_-Ca_2_Al, H_1_M_1_-Ca_2_Al, H_1_M_2_-Ca_2_Al, H_1_M_3_-Ca_2_Al), the (002) reflection peaks appear at ca. 3.4°, corresponding to the *d*-spacing values of 2.55, 2.68, 2.55, and 2.75 nm, respectively. The enlarged *d*-spacing of H*_n_*M*_n_*_′_-Ca_2_Al-LDHs suggests that HALS and MP anions were co-intercalated into the LDH, and the different ratios of HALS/MP result in a slightly different arrangement of guest anions leading to minor variations of the *d*-spacing values. The full width at half maximum values of the (002) reflection of all H*_n_*M*_n_*_′_-Ca_2_Al compounds are smaller than those of HALS-Ca_2_Al and MP-Ca_2_Al, indicating that the number of stacked platelets was decreased due to the co-intercalation. The results show that co-precipitation yields Ca_2_Al-LDHs free of CaCO_3_ by-product [23].

**Figure 3:**
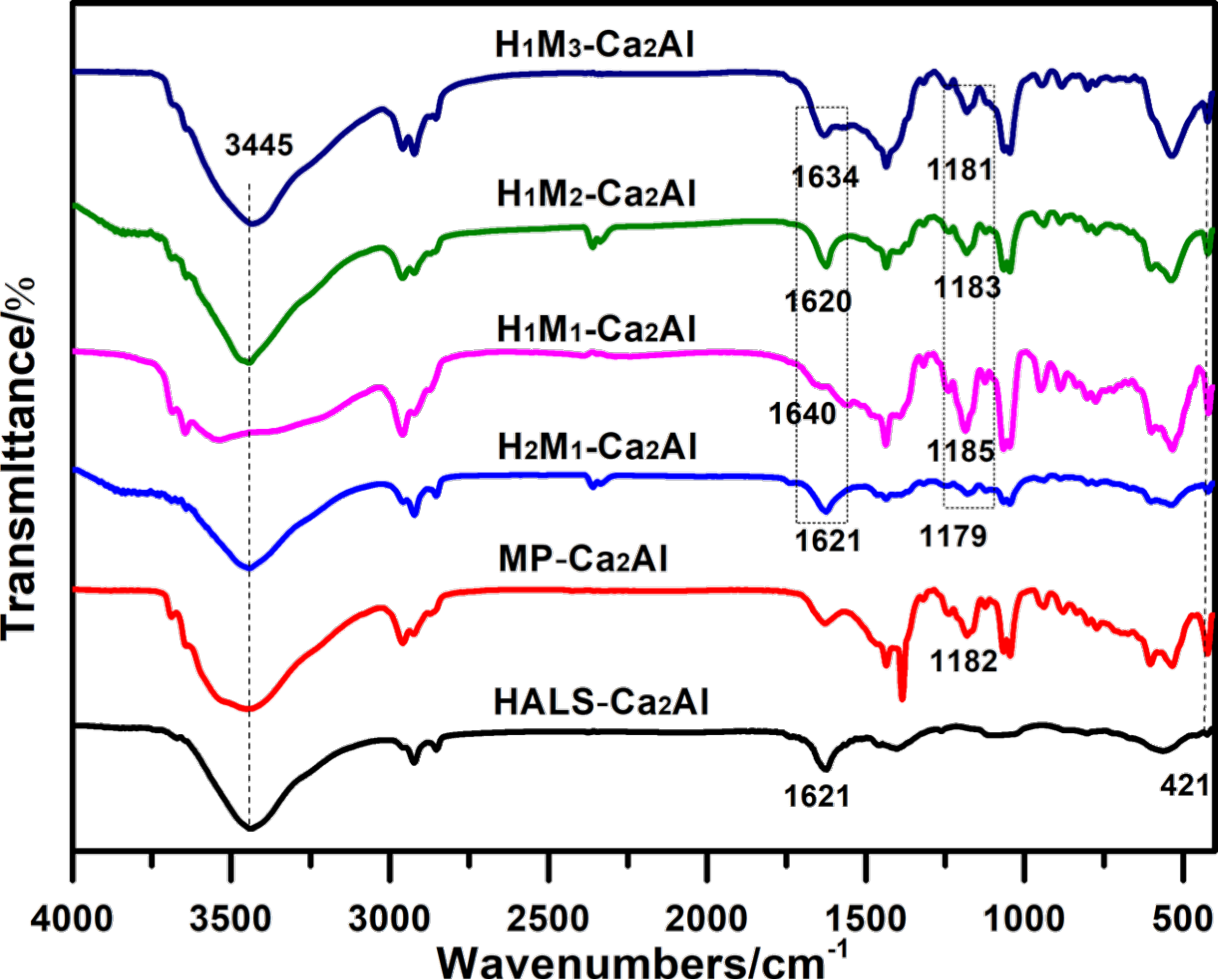
FTIR spectra of different H*_n_*M*_n_*_′_-Ca_2_Al-LDH samples.

Figure 3 shows FTIR spectra of all the H*_n_*M*_n_*_′_-Ca_2_Al-LDHs. One can observe characteristic stretching-vibration bands of LDHs, for example, the broad band at ca. 3445 cm^−1^ associated to the OH groups of interlayer water molecules and brucite-like LDH layers. The band at 421 cm^−1^ is attributed to O–M–O lattice vibrations in LDH, which further proves the formation of a LDH platelet structure. Moreover, for HALS-Ca_2_Al-LDH and MP-Ca_2_Al-LDH, the characteristics stretching vibration bands of HALS and MP also occur, such as the carbonyl group of HALS at 1621 cm^−1^ (C=O) and the phosphate group of MP at 1181 cm^−1^ (P=O), 1050 cm^−1^ (P–O–C). Compared with 1645 cm^−1^ in HALS and 1164 cm^−1^ in MP-Ca, shifts of C=O and P=O are observed to 1621 cm^−1^ in HALS-Ca_2_Al and 1182 cm^−1^ in MP-Ca_2_Al, respectively, which probably results from the electrostatic interaction between the organic anions and the host sheets of Ca_2_Al-LDH. After co-intercalation of HALS and MP, H*_n_*M*_n_*_′_-Ca_2_Al samples demonstrate all of the characteristic absorption bands of the LDH host together with those of HALS and MP, suggesting the coexistence of active HALS and MP species within H*_n_*M*_n_*_′_-Ca_2_Al-LDH.

**Figure 4:**
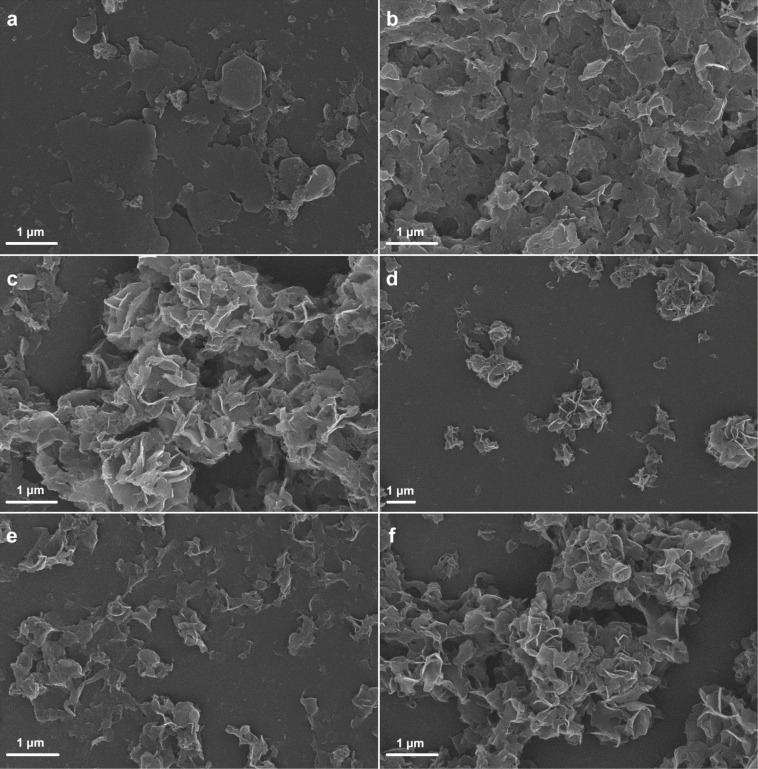
SEM images of (a) HALS-Ca_2_Al, (b) MP-Ca_2_Al and (c) H_2_M_1_-Ca_2_Al, (d) H_1_M_1_-Ca_2_Al, (e) H_1_M_2_-Ca_2_Al, (f) H_1_M_3_-Ca_2_Al.

Figure 4 presents SEM images of HALS-Ca_2_Al, MP-Ca_2_Al and H*_n_*M*_n_*_′_-Ca_2_Al-LDHs. HALS-Ca_2_Al and MP-Ca_2_Al show typical platelet-like morphologies. In comparison with MP-Ca_2_Al, HALS-Ca_2_Al exhibits a flattened platelet-like structure and a larger average particle size. For the co-intercalated H*_n_*M*_n_*_′_-Ca_2_Al, one observes a significant aggregation of LDH platelets leading to a porous flower-like morphology.

**Figure 5:**
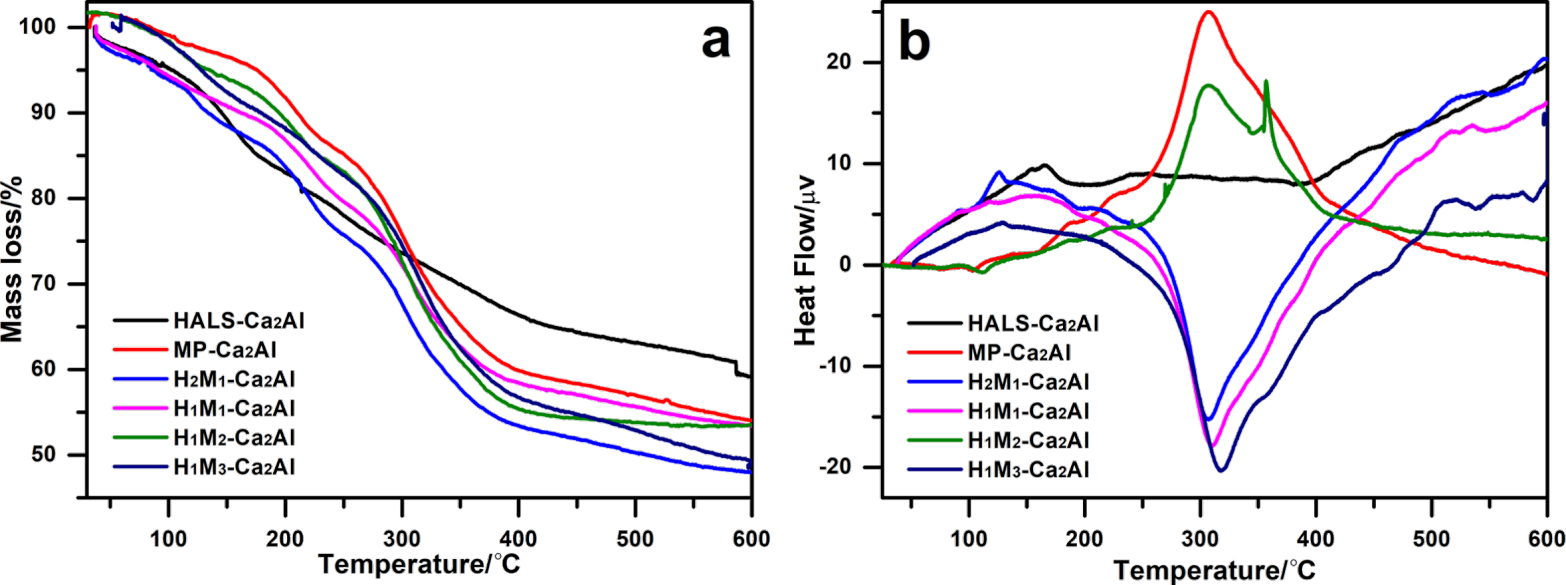
(a) TG and (b) DTA curves of Ca_2_Al-LDHs: HALS-Ca_2_Al, MP-Ca_2_Al, H_2_M_1_-Ca_2_Al, H_1_M_1_-Ca_2_Al, H_1_M_2_-Ca_2_Al, and H_1_M_3_-Ca_2_Al.

Figure 5 shows the TG and DTA curves of H*_n_*M*_n_*_′_-Ca_2_Al-LDHs and Table 1 summarizes the corresponding data. In our previous work, the decomposition of HALS and Irganox 1425 molecular anions occurred with an exothermic DTA peak at 300 and 295 °C, respectively [16–17]. Here, three major stages of mass loss in the TG curve of H*_n_*M*_n_*_′_-Ca_2_Al-LDH samples can be observed. The first mass loss up to 180 °C is assigned to the release of adsorbed water and crystal water; The second one in the range of 180–250 °C is attributed to the dehydroxylation of the metal-hydroxide layer. The third large mass loss stage corresponding to the decomposition of HALS and MP ions appears at 250–450 °C with endothermic peaks between 300 and 360 °C in the DTA curve. The thermal stability of H*_n_*M*_n_*_′_-Ca_2_Al-LDHs was expressed through the temperatures associated to a certain weight loss (i.e., *T*_25%_ is the temperature at which the sample has lost 25 wt %) in Table 1. For intercalated Ca_2_Al-LDHs, the thermal oxidative decomposition occurs at temperatures higher than those of HALS and Irganox 1425. Moreover, the co-intercalated H*_n_*M*_n_*_′_-Ca_2_Al-LDHs exhibit a higher decomposition temperature than HALS-Ca_2_Al and MP-Ca_2_Al, especially H_1_M_2_-Ca_2_Al (356 °C). For the co-intercalated H*_n_*M*_n_*_′_-Ca_2_Al-LDHs, the T_25%_ values gradually increase from 257 °C for H_2_M_1_-Ca_2_Al to 299 °C for H_1_M_3_-Ca_2_Al with an increasing content of M. The above results illustrate that the thermal stability of HALS and MP anions are enhanced after the co-intercalation of both anions into the interlayer region of LDHs.

**Table 1:** TG/DTA results of H*_n_*M*_n_*_′_-Ca_2_Al-LDH samples.

sample	*T*_25%_ (°C)	DTA peak (°C)	residual mass (wt %)

HALS-Ca_2_Al	283	300	55
MP-Ca_2_Al	303	308	51
H_2_M_1_-Ca_2_Al	257	310	45
H_1_M_1_-Ca_2_Al	286	312	49
H_1_M_2_-Ca_2_Al	293	306, 356	52
H_1_M_3_-Ca_2_Al	299	320	48

Table 2 lists the element analysis data and the calculated chemical compositions of H*_n_*M*_n_*_′_-Ca_2_Al-LDHs analyzed by CHN elemental analysis for the organic moieties and ICP atomic emission spectrometry for metal cations. The content of interlayer water is determined from the mass loss between 100 and 200 °C in the TG curves (Figure 5a). The fractions of HALS and MP anions are calculated based on the content of Al and C taking into account the charge balance. The molar fractions of the guest anions are close to the feeding ratio, suggesting the ratio between HALS and MP can be adjusted as designed. These results also suggest that both of HALS and MP anions have been co-intercalated into Ca_2_Al-LDH.

**Table 2:** Chemical composition of H*_n_*M*_n_*_′_-Ca_2_Al-LDH samples.

sample	Ca (wt %)	Al (wt %)	C (wt %)	Ca/Al	H_2_O (wt %)	chemical composition

HALS-Ca_2_Al	14.4	5.1	28.6	2.0	12.2	Ca_0.67_Al_0.33_(OH)_2_(HALS)_0.33_·1.1H_2_O
MP-Ca_2_Al	12.9	3.5	31.4	2.1	7.3	Ca_0.68_Al_0.32_(OH)_2_(MP)_0.32_·0.85H_2_O
H_2_M_1_-Ca_2_Al	15.8	3.8	29.1	2.3	8.4	Ca_0.7_Al_0.3_(OH)_2_(HALS)_0.2_(MP)_0.1_·0.68H_2_O
H_1_M_1_-Ca_2_Al	14.5	3.7	30.2	2.2	7.5	Ca_0.69_Al_0.31_(OH)_2_(HALS)_0.15_(MP)_0.16_·0.72H_2_O
H_1_M_2_-Ca_2_Al	14.9	3.3	31.6	2.3	9.4	Ca_0.7_Al_0.3_(OH)_2_(HALS)_0.1_(MP)_0.2_·0.83H_2_O
H_1_M_3_-Ca_2_Al	16.5	3.9	29.5	2.5	8.0	Ca_0.72_Al_0.28_(OH)_2_(HALS)_0.07_(MP)_0.21_·0.66H_2_O

### Analysis of H*_n_*M*_n_*_′_-Ca_2_Al-LDHs/PP composites

**Figure 6:**
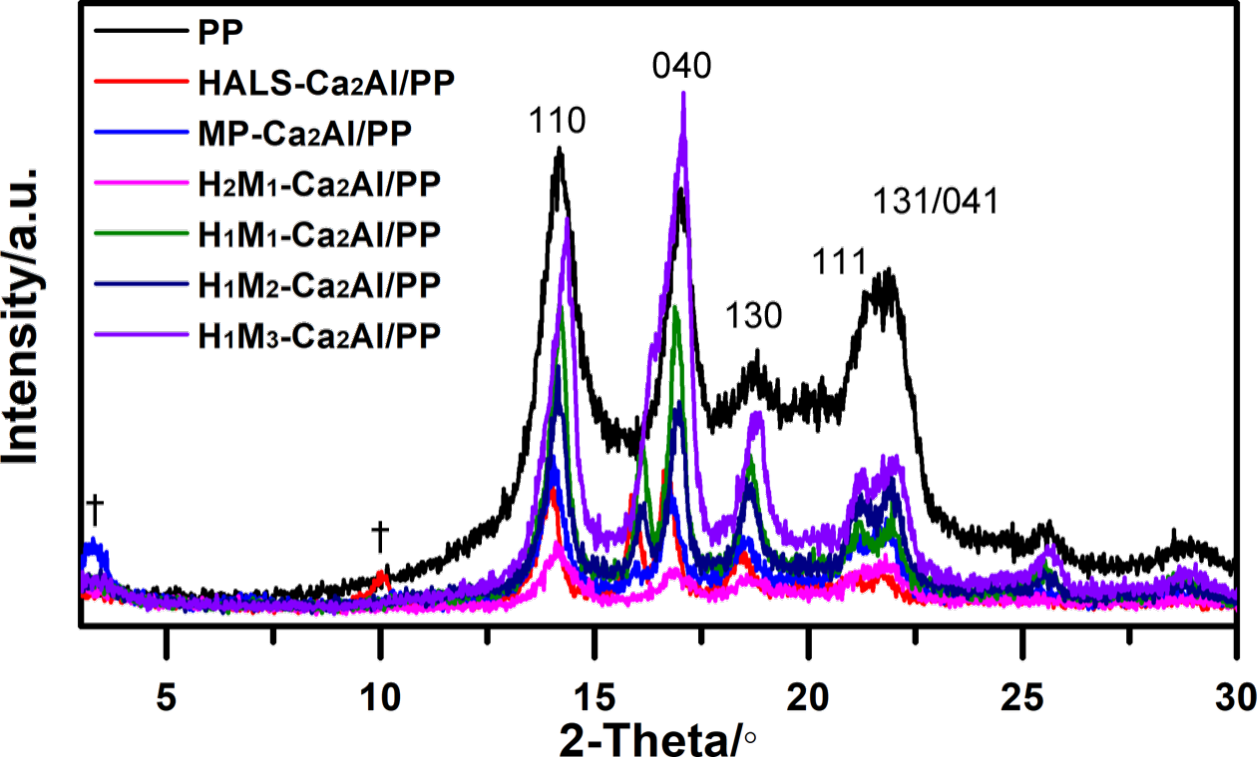
Powder X-ray diffraction pattern of H*_n_*M*_n_*_′_-Ca_2_Al/PP composites. LDH reflection peaks were marked with “†”.

Figure 6 shows XRD patterns of H*_n_*M*_n_*_′_-Ca_2_Al-LDHs/PP composites. All samples show the characteristic Bragg reflections of α-PP at 12–30° for (110), (040), (130), (111) and (131)/(041) *d*-spacings. That is, the addition of H*_n_*M*_n_*_′_-Ca_2_Al-LDHs has only insignificant influence on the crystallization behavior of PP [24]. For HALS-Ca_2_Al/PP and MP-Ca_2_Al/PP, the typical reflection (002) peaks of HALS-Ca_2_Al and MP-Ca_2_Al clearly appear (marked with “†”) with an increase in spacing from 0.77 to 0.88 nm for HALS-Ca_2_Al and from 2.52 to 2.68 nm for MP-Ca_2_Al. Probably, the PP chains were intercalated into the LDH gap structure to produce a polymer-intercalated nanocomposite [25]. Yet, none of the diffraction peaks of the co-intercalated H*_n_*M*_n_*_′_-Ca_2_Al hybrid LDHs is observed in the resulting H*_n_*M*_n_*_′_-Ca_2_Al/PP composites, suggesting a high dispersion of LDH nanoparticles in the composite.

**Figure 7:**
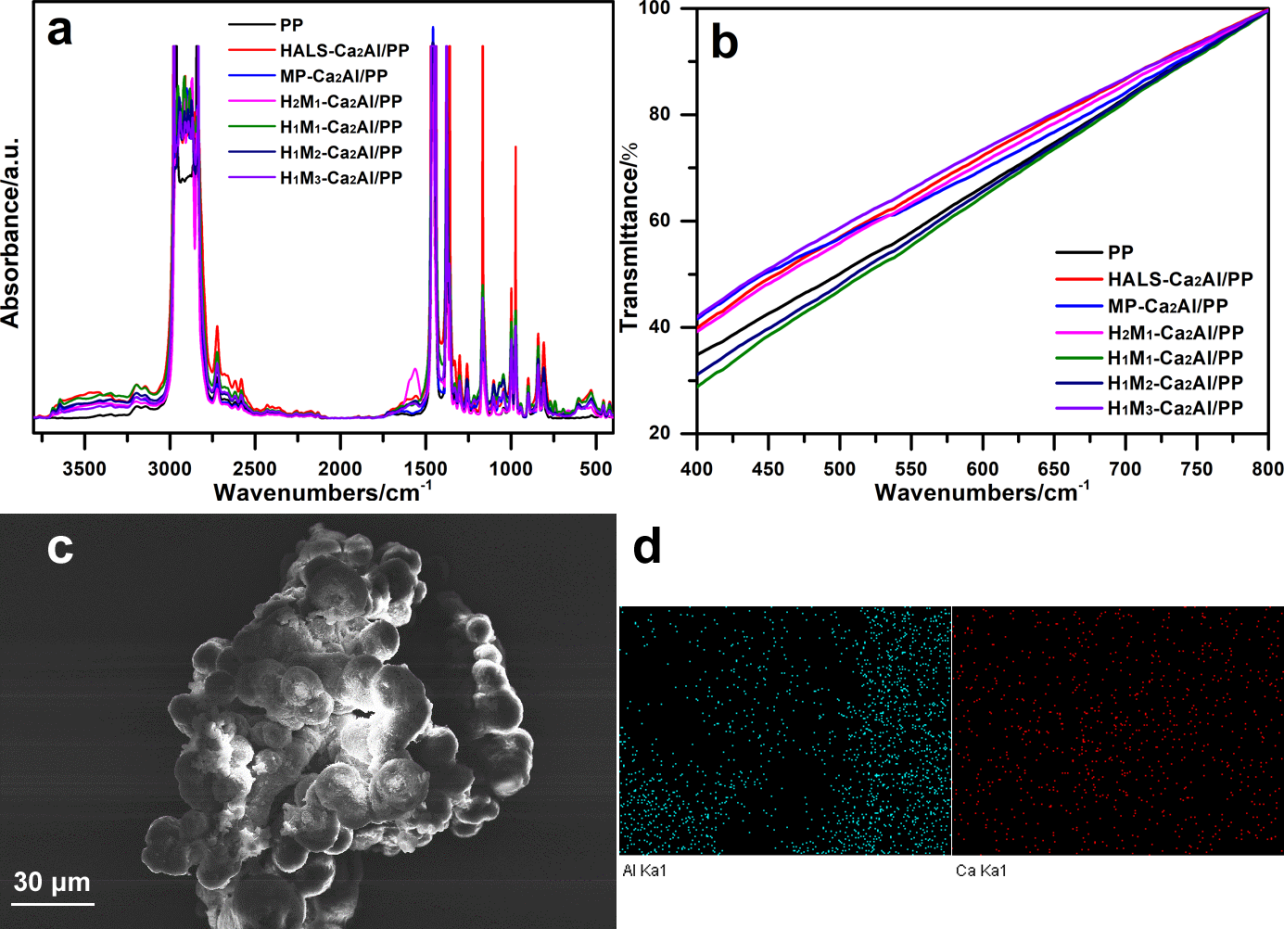
(a) FTIR spectra and (b) visible-light transmittance spectra of Ca_2_Al/PP composites. (c) SEM image and (d) Al (left) and Ca (right) element mapping of the H_1_M_1_-Ca_2_Al/PP composite.

Figure 7a depicts the FTIR spectra of H*_n_*M*_n_*_′_-Ca_2_Al/PP composites in absorbance mode. Here, all composites present the characteristic bands of PP: 2950, 2915, 2868, 2837, 1454, and 1375 cm^−1^. Some additional bands assigned to LDHs and guest anions are also observed after addition of H*_n_*M*_n_*_′_-Ca_2_Al-LDHs. Figure 7b demonstrates the visible-light transmittance of H*_n_*M*_n_*_′_-Ca_2_Al-LDH/PP composite films, which is one of crucial properties of the PP products. All the samples show a similar trend demonstrating that there is a good dispersion of Ca_2_Al-LDHs in the PP matrix without affecting its visible-light transmission. Figure 7c,d displays the surface morphology and element distribution of H_1_M_1_-Ca_2_Al/PP composites from SEM and element mapping. Consistent with PP free of filler, a spherical structure is observed for Ca_2_Al/PP composites and Al (left) and Ca (right) elements are homogenously distributed in the H_1_M_1_-Ca_2_Al/PP composite films. All results confirm that Ca_2_Al-LDH particles are well dispersed in the PP matrix and have no negative effect on the structure and morphology.

### Performance of H*_n_*M*_n_*_′_-Ca_2_Al/PP composites

**Figure 8:**
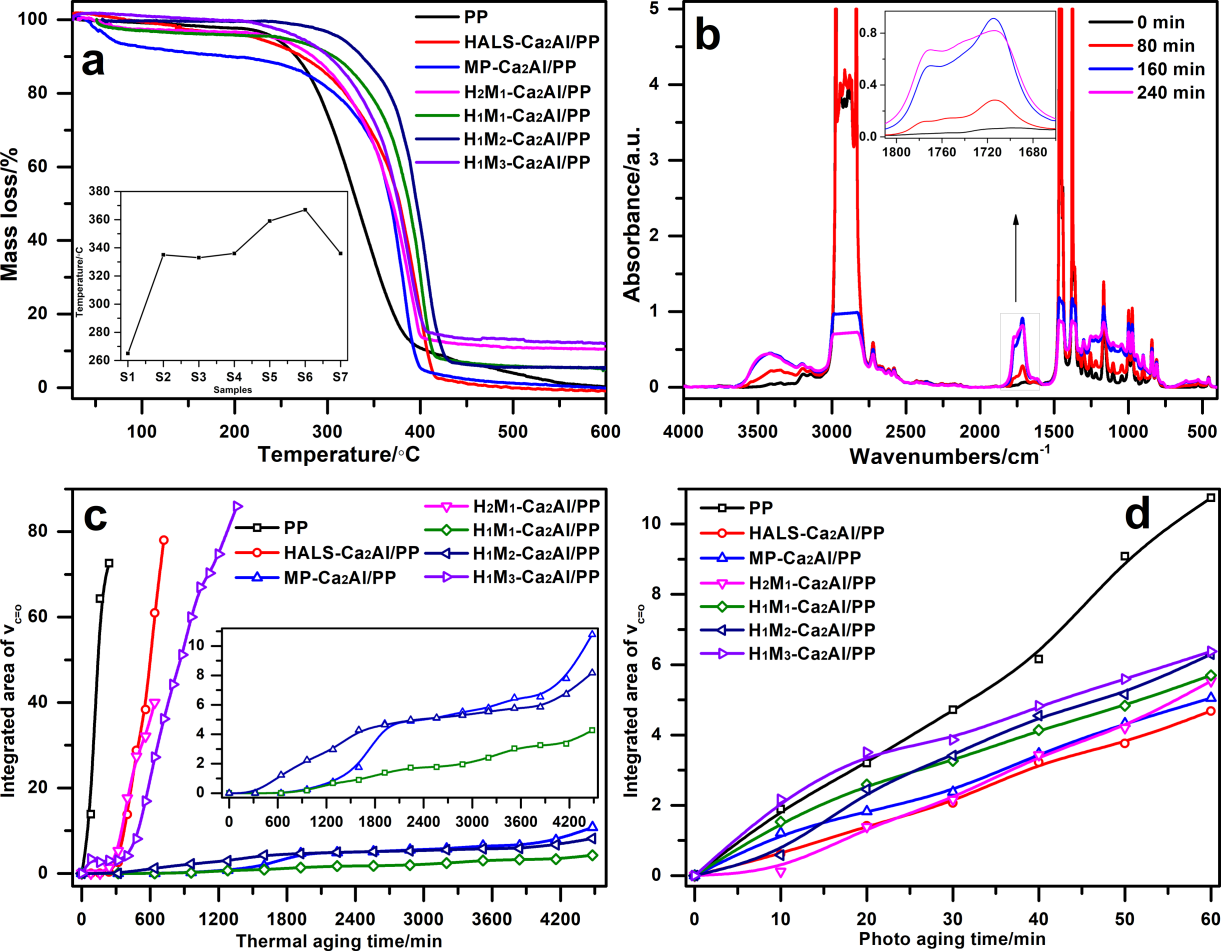
(a) TGA curves of H*_n_*M*_n_*_′_-Ca_2_Al/PP composites. (b) FTIR spectra of PP after different periods of thermal aging. (c) Thermal aging and (d) photo-oxidation aging of H*_n_*M*_n_*_′_-Ca_2_Al/PP films.

Figure 8a shows the thermal decomposition of H*_n_*M*_n_*_′_-Ca_2_Al/PP composites measured by TG-DTA. The thermal decomposition observed for Ca_2_Al/PP composites is comparable to that of PP free of filler, and the main decomposition process for all samples occurs between 250 and 450 °C. The incorporation of H*_n_*M*_n_*_′_-Ca_2_Al-LDHs is found to increase the onset temperature (*T*_onset_) of the initial degradation process. The onset values for co-intercalated H*_n_*M*_n_*_′_-Ca_2_Al/PP composites are higher *T*_onset_ in the range of 336–367 °C than those of PP free of filler (265 °C), HALS-Ca_2_Al/PP (335 °C) and MP-Ca_2_Al/PP (333 °C). However, the onset temperature of co-intercalated H*_n_*M*_n_*_′_-Ca_2_Al/PP does not increase with the percentage of M. H_2_M_1_-Ca_2_Al/PP has the highest *T*_onset_ value. Moreover, with the addition of H*_n_*M*_n_*_′_-Ca_2_Al-LDHs, the amount of residue is also increased, the promotion of the carbonization process leads to a better flame retardancy of Ca_2_Al/PP composites. As a result, the thermal stability of H*_n_*M*_n_*_′_-Ca_2_Al/PP composites is obviously improved.

Figure 8b shows the thermal degradation of H*_n_*M*_n_*_′_-Ca_2_Al/PP composite films at 150 °C as a function of aging time, recorded by FTIR. With increasing thermal-aging time, the intensity of the carbonyl peak of the PP film (without filler) significantly increases in the range of 1810–1660 cm^−1^, accompanied by changes in shape and position. Here, the integrated area of the carbonyl band is measured to quantitatively analyze the degradation degree of H*_n_*M*_n_*_′_-Ca_2_Al/PP composite films. Figure 8c shows the integrated area as a function of the aging time. Two things can be noted: (1) PP, HALS-Ca_2_Al/PP, H_2_M_1_-Ca_2_Al/PP and H_1_M_3_-Ca_2_Al/PP films, during rapid thermal aging, completely break after less than 1600 min. (2) MP-Ca_2_Al/PP, H_1_M_1_-Ca_2_Al/PP and H_1_M_2_-Ca_2_Al/PP films exhibit a good stability against thermal aging, and their appearance remains intact after 4500 min at 150 °C. The additive MP-Ca is an excellent protection against thermal degradation, while HALS is a photo-oxidation stabilizer. With the same amount of H and M, the composite H_1_M_1_-Ca_2_Al/PP shows the best thermal stability among all intercalated Ca_2_Al-LDHs. Also, the ratio between HALS and MP can be used to slightly tune the thermal properties of the LDH/PP composites.

Figure 8d displays the photo-oxidation degradation of H*_n_*M*_n_*_′_-Ca_2_Al/PP composite films under UV irradiation. The integrated area in the range of 1810–1660 cm^−1^ for all samples becomes larger with increasing UV exposure time. In comparison with PP free of filler, the addition of H*_n_*M*_n_*_′_-Ca_2_Al-LDHs enhances the photo-oxidation stability. The photo-oxidation stability is in the following order: HALS-Ca_2_Al/PP > H_2_M_1_-Ca_2_Al/PP > MP-Ca_2_Al/PP > H_1_M_1_-Ca_2_Al/PP > H_1_M_2_-Ca_2_Al/PP > H_1_M_3_-Ca_2_Al/PP. The different co-intercalated H*_n_*M*_n_*_′_-Ca_2_Al-LDHs are found to enhance the thermal and photo-oxidation stability of H*_n_*M*_n_*_′_-Ca_2_Al/PP composite films, and the co-intercalated LDH/PP composite films have better overall performances compared with the systems intercalated with HALS or MP only.

## Conclusion

In this work, we have successfully co-intercalated a hindered amine light stabilizer (HALS) and a hindered phenolic antioxidant (MP) into the interlayer region of Ca_2_Al-LDHs with different molar ratios through coprecipitation. The concomitant intercalation of HALS and MP significantly enhances the thermal stability of the powders due to the host–guest interactions between guest anions and the host LDH. Subsequently a series of H*_n_*M*_n_*_′_-Ca_2_Al/PP composite films was prepared. The results show that the addition of H*_n_*M*_n_*_′_-Ca_2_Al-LDH has no negative effect on the crystallization behavior of PP, while it improves significantly the stability of the composites against thermal degradation and photo-oxidation. Undoubtedly, the co-intercalation method for LDH framework will open a way to design and fabricate multifunctional additives for polymer composites.

## Experimental

### Chemicals

Succinic anhydride, tetramethylpiperidinamine, dioxane, ether, Ca(NO_3_)_2_·4H_2_O, Al(NO_3_)_3_·9H_2_O, NaOH, C_2_H_5_OH, CH_3_COCH_3_, xylene and hexane were directly used as received from Beijing Chemical Co. Limited. Deionized water was employed in all experiments. Polypropylene (PP1300, melting index: 1.5 g/10 min; melting point: 164–170 °C; density: 0.91 g·cm^−3^), and Irganox 1425 were supplied from Beijing Yanshan Petrochemical Co. Ltd., China.

### Fabrication of HALS

The HALS was synthesized as reported [16]. Typically, succinic anhydride (15 mmol) was dissolved into 10 mL of dioxane at 80 °C under vigorous stirring, and tetramethylpiperidinamine (15 mmol) in 10 mL of dioxane was dropwise added. The solution was kept at 80 °C for 40 min. The product was washed three times using dioxane and ether. Finally, the powdered product HALS was collected after vacuum filtration.

### Fabrication of H*_n_*M*_n_*_′_-Ca_2_Al-LDHs

The HALS and MP co-intercalated LDHs (H*_n_*M*_n_*_′_-Ca_2_Al-LDHs) were prepared through coprecipitation with different H/M molar ratios of 2:1, 1:1, 1:2, 1:3. For H_1_M_1_-Ca_2_Al, HALS (3.072 g, 12 mmol) and Irganox 1425 (4.17 g, 6 mmol) were dissolved in 240 mL of ethanol/water (3:1, v/v). A solution containing 0.100 mol·L^−1^ Al(NO_3_)_3_·9H_2_O and 1.40 mol·L^−1^ NaOH was added dropwise to the above HALS/MP-Ca solution at room temperature under vigorous stirring in nitrogen atmosphere. The pH value in the reaction system was maintained at pH 10 after finishing the addition, and the reaction was kept for another 12 h. The suspension was centrifuged and washed with 60% ethanol solution until pH 7. The resulting slurry was further washed twice with acetone with surface modification and then was used for the preparation of H_1_M_1_-Ca_2_Al/PP composites. To obtain the H_1_M_1_-Ca_2_Al powder, part of the slurry was dried in an oven at 80 °C for 24 h. Co-intercalated H*_n_*M*_n_*_′_-Ca_2_Al with different molar ratios and MP-Ca_2_Al were obtained through a similar process. Besides, HALS-Ca_2_Al as the reference was prepared similarly with a metal solution of Ca(NO_3_)_2_·4H_2_O and Al(NO_3_)_3_·9H_2_O.

### Fabrication of H*_n_*M*_n_*_′_-Ca_2_Al/PP composites

A series of H*_n_*M*_n_*_′_-Ca_2_Al/PP composites was fabricated through solvent mixing with the same mass loading of 4.0 wt % compared with pure PP [26]. For the example of H_1_M_1_-Ca_2_Al/PP, 6.36 g of H_1_M_1_-Ca_2_Al slurry (solid content: 6.30 wt %) was dispersed in 100 mL xylene containing 10.0 g PP and the suspension was heated to 140 °C in an oil bath under vigorous stirring for 3 h. The resulting suspension was immediately transferred into 50 mL hexane solvent and then cooled down to 25 °C. Finally, the solid product was collected after drying to constant weight at 80 °C. For further analyses, the H_1_M_1_-Ca_2_Al/PP composite was pressed into a composite film by Teflon sheets at 170 °C and the thickness was controlled to be ca. 0.1 mm. Composites of other LDHs with PP (HALS-Ca_2_Al/PP, MP-Ca_2_Al/PP, H_2_M_1_-Ca_2_Al/PP, H_1_M_2_-Ca_2_Al/PP, H_1_M_3_-Ca_2_Al/PP) were prepared following a similar process using the required amount of LDH slurry.

### Characterization

Powder X-ray diffraction (XRD) measurements were performed on a Shimadzu XRD-6000 X-ray diffractometer with a wavelength of 0.154 nm at 40 kV and 30 mA in a 2θ range of 3–70° at 10°·min^−1^. Fourier-transform infrared (FTIR) spectra were recorded on a Bruker Vector 22 infrared spectrophotometer with KBr pellets (sample/KBr of 1:100 by weight) or thin films. Thermogravimetry and differential thermal analysis (TG-DTA) was performed on a PCT-IA instrument in the range of 25 to 700 °C at 5 °C·min^−1^ under flowing air. Scanning electron microscopy (SEM) images were taken with a Zeiss scanning electron microscope by dropping dilute ethanol suspension at room temperature. Elemental analysis for metal elements (Ca and Al) was carried out on a Shimadzu ICPS-7500 inductively coupled plasma (ICP) atomic emission spectrometer. About 30 mg of the samples was dissolved in a few drops of concentrated nitric acid (65%) and diluted to 10 mL using water. CHN elemental analysis was carried out on a Vario EL III, Elementar instrument. The content of water in the samples was obtained by thermogravimetry. The UV–vis spectra in the range of 200 to 800 nm were collected by using a Shimadzu UV-2501PC spectrophotometer.

### Stability evaluation of H*_n_*M*_n_*_′_-Ca_2_Al/PP composites

Here, two methods were employed to evaluate the thermal stability of H*_n_*M*_n_*_′_-Ca_2_Al/PP composites. One way was to examine the composite samples with TG-DTA, for example, ca. 7 mg of the samples was heated from 25 to 600 °C at 10 °C·min^−1^ in flowing air. The other was to perform an accelerated thermal aging test in an oven [15]. For this, H*_n_*M*_n_*_′_-Ca_2_Al/PP composite films were tailored to a size of 20 × 20 × 0.1 mm and thermally aged at 150 °C. Every 80 min, the composition was monitored by FTIR. For the quantitative analysis of the degradation, the integrated area of peaks in the range of 1810–1660 cm^−1^, assigned to carbonyl groups was used.

The photo stability of H*_n_*M*_n_*_′_-Ca_2_Al/PP composites (20 × 20 × 0.1 mm) was examined in an accelerated photo-aging instrument with an ultraviolet high-pressure mercury lamp (*P* = 100 W, λ_max_ = 360 nm) and the degradation degree was monitored every 5 min by FTIR [27]. The data processing method was the same as during the thermal aging.
